# Towards an off-grid fecal sludge treatment unit: demonstrating energy positive thermal treatment

**DOI:** 10.12688/gatesopenres.12929.1

**Published:** 2019-04-10

**Authors:** Taylor Myers, Lars Schoebitz, Stuart Woolley, Jaime Sanchez Ferragut, Jimmy Thostenson, Kathy Jooss, Jeffery Piascik, August Frechette, Nico Hotz, Brian R. Stoner, Jeffery Hallowell

**Affiliations:** 1 Biomass Controls, Putnam, CT, 06260, USA; 2 Duke University, Durham, NC, 27708, USA

**Keywords:** fecal sludge, fecal sludge treatment, thermochemical, energy independence, energy neutrality, energy positivity, thermal, fecal sludge treatment unit, energy generation

## Abstract

**Background**: There is an unmet demand for community-scale fecal sludge treatment units (FSTUs) that serve communities of between 1,000 and 50,000 people and are able to operate in non-sewered and off-grid environments. An emerging industry standard for FSTUs includes as a key criteria energy independence in steady-state. Theoretically, there is sufficient thermal energy available in fecal sludge to provide the electrical power needed to run the FSTU. However, such a system had never been implemented.

**Methods**: Biomass Controls has previously demonstrated the thermal treatment of fecal sludge using the Biogenic Refinery, a thermal FSTU deployed in three sites in India. In this article we describe testing where a Biogenic Refinery was paired with a thermal fluid heat exchanger and organic Rankine cycle generator to generate electrical power.

**Results**: This Biogenic Refinery combined heat and power system generated sufficient electrical power to offset electrical parasitic loads in steady-state operation and produce a surplus of 1.2 kWe.

**Conclusions**: The results of the study demonstrate that there is an excess of energy available and reliable mechanisms to generate electrical energy using an FSTU. Additional steps are necessary to transition to a true off-grid FSTU.

## Introduction

Community scale fecal sludge treatment units (FSTUs) are an important technology in meeting the needs of the 2.5 billion people without adequate sanitation ( Rose *et al*., 2015; WHO/UNICEF, 2017). Many of these communities also lack access to consistent electricity supply ( IEA and Bank, 2019). To encourage the development of FSTUs that meet these needs, the International Standards Organization (ISO) is developing the ISO/PC318 standard for community scale resource oriented sanitation treatment systems ( ISO/PC318, 2019). Key criteria for compliance with this emerging standard include: (1) the inactivation of pathogens present in fecal sludge and (2) energy independence in steady-state operation. The first key criteria may be met through a variety of different treatment techniques including thermal treatment, high pH processes, composting, irradiation, and aerobic digestion. To meet the second key criteria, the treatment process must be energy neutral or energy positive, and operate on faecal sludge only without supplemental energy from other sources.

Thermal treatment technologies are a promising approach that could meet both of these criteria ( Onabanjo *et al*., 2016; Yermán *et al*., 2015). In contrast to other pathogen elimination strategies, high temperature thermal treatment offers rapid inactivation of pathogens in a small footprint, without requiring disinfecting chemicals. Further, thermal treatments, including pyrolysis, gasification, and combustion, can liberate stored energy present in the fecal sludge providing thermal energy useful for processing the fecal sludge and for conversion to electrical energy to run the process ( Syed-Hassan *et al*., 2017).

This paper focuses on the demonstration of energy independence in steady-state operation, which is a key criteria for compliance with emerging standard ISO/PC318. In the present work, the Biomass Controls Biogenic Refinery (BR), an existing thermal FSTU, was paired with a thermal fluid heat exchanger and organic Rankine cycle (ORC) generator to make a combined heat and power (CHP) unit. The BR is currently used as an FSTU in several communities in India fulfilling pathogen inactivation requirements given in Environmental Protection Agency (EPA) 40 CFR Part 503 ( US EPA, 2018). The objective of the present study is to demonstrate that there is sufficient electrical power produced by the BR CHP to offset the electrical power necessary to run the BR CHP.

To provide this demonstration, first, we describe the BR CHP system and its modules as well as the test protocol followed. Next, we present a thermal power balance based on measurements of the system during steady-state operation to provide energy available in the thermochemical process. Last, we present measurements of electrical power generated by the ORC, as well as the electrical power consumed by the BR CHP system to calculate net power produced. The result of the study is a demonstration that the key requirements for the emerging standard for community scale FSTUs are achievable.

## Methods

### Description of the Biogenic Refinery

The BR CHP system consists of four connected modules: (1) the pyrolysis-combustion module responsible for pyrolysing the feedstock and liberating thermal energy from the feedstock; (2) the oil working fluid heat exchanger module responsible for directing a portion of the available thermal energy towards the ORC module; (3) the ORC module responsible for converting thermal energy to electricity; and (4) the hydronic heat exchanger module responsible for extracting additional thermal energy for the purpose of drying incoming feedstock.

The BR system is a thermal FSTU capable of refining any biogenic matter into inert carbon with significant volume reduction, carbon sequestration in the form of biochar, and controlled emissions. The BR uses a pyrolysis-combustion process to inactive pathogens while producing thermal energy and biochar, useful as an agricultural soil amendment, as outputs ( Jeffery *et al*., 2011). The BR is capable of processing approximately 150 kg dry basis of feedstock per day (based on 8 hours of operation), consistent with the ISO/PC318 goal of serving small communities. This amounts to a >18 kg/hr (dry basis) average across a daily 8 hour process period.

Thermal energy from the feedstock is released by the pyrolysis-combustion module and exits in hot exhaust gases. This thermal energy is extracted from the hot exhaust gases by a heat exchanger filled with a thermal working fluid, in our case oil. The thermal energy from the oil working fluid is transferred to the ORC module. The thermal energy remaining in the hot exhaust gases is transferred by the hydronic heat exchanger to further heat the water which is later used for drying.

### Description of ORC electrical generation

**Figure 1.**
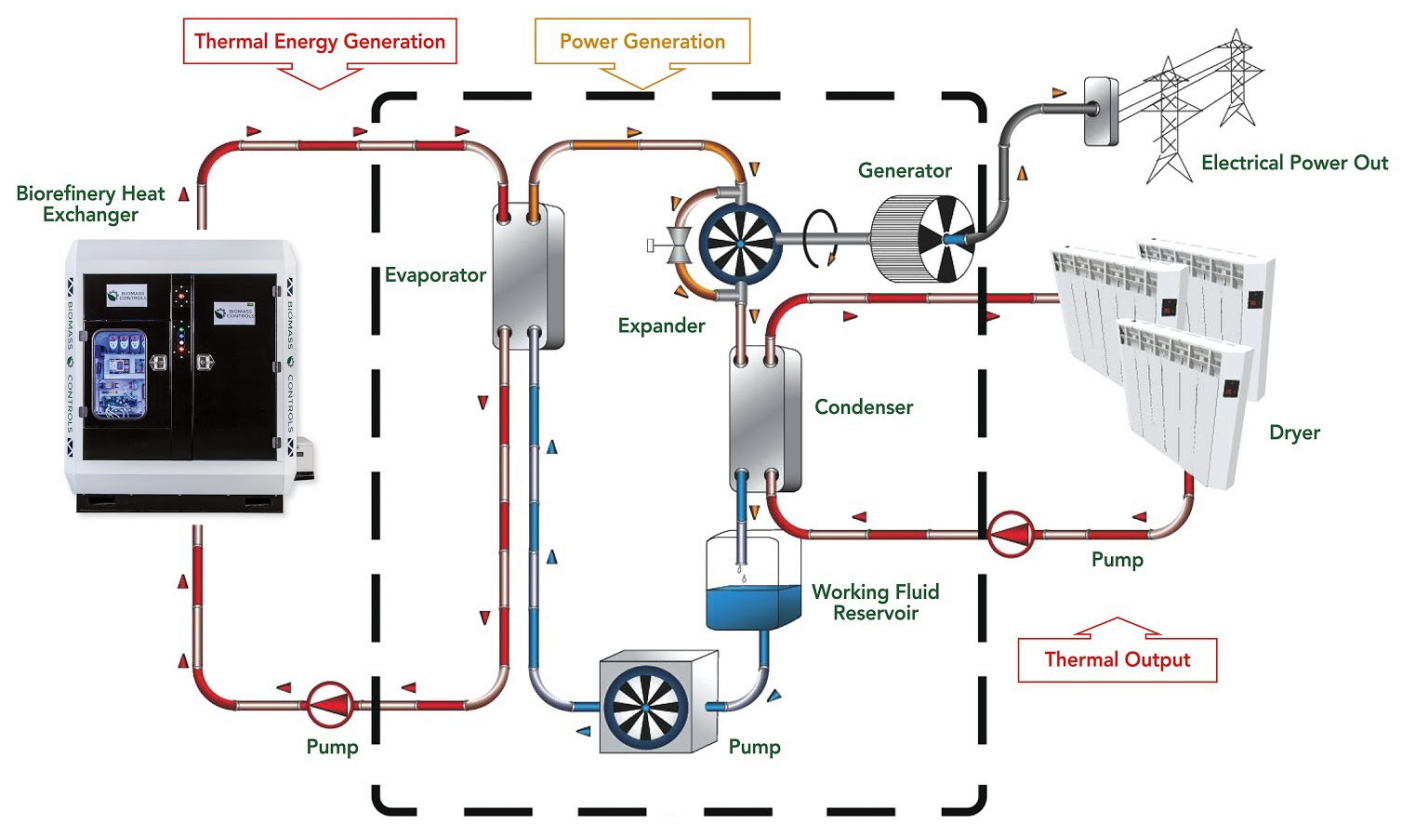
The Biogenic Refinery Combined Heat and Power system uses thermal energy produced through the thermal treatment of feedstocks to generate electrical power using an organic Rankine cycle generator (black dashed box).

The ORC module uses the thermal energy from the BR to generate electricity. A schematic of the BR CHP system operation can be seen in Figure 1.

The thermal energy is delivered from the BR to the ORC module using an oil working fluid (Duratherm FG by Duratherm Extended Life Fluids, Lewiston, USA). This working fluid is used to evaporate a different organic working fluid within the ORC. This evaporated fluid is used in conjunction with a scroll expander and runs an electric generator. Following electrical generation, the working fluid is condensed using a water heat exchanger within the ORC, and the heated water is used for heating. The ORC used in the BR CHP system had a rated electrical generation capacity of 4 kWe with a nominal 8% generation efficiency. Because the ORC has only 8% efficiency in generating electricity, 92% of the original thermal energy remains available for heating applications following energy conversion. In the BR CHP, this energy is used to heat water which is then further heated by the BR hydronic heat exchanger module and ultimately used for the drying of incoming feedstock.

### Feedstock

FSTUs are intended to primarily treat fecal sludge. However, in a laboratory setting, a sufficient volume of fecal sludge for extended testing is difficult to acquire. Instead, a surrogate feedstock with a similar caloric value, or effective heat of combustion, was sought. The effective heat of combustion of dry feces varies substantially depending on location and source. A study by Gold *et al*. (2017) found effective heats of combustion of 13.4 MJ/kg and 10.9 MJ/kg in Dakar, Senegal and Kampala, Uganda, respectively. A study by Muspratt *et al*. (2014) found effective heats of combustion of 16.6 MJ/kg, 16.2 MJ/kg, and 19.1 MJ/kg in Dakar, Kampala, and Kumasi, Ghana, respectively. A recent study by Myers *et al*. (n.d.) found effective heats of combustion of 19.6 MJ/kg and 22.3 MJ/kg in samples from India and the United States, respectively. A meta-study by Rose *et al*. (2015) found the average and median for dry feces internationally are 17.2 MJ/kg and 19.1 MJ/kg. Wood pellets (Tractor Supply Company SKU # 3195163) with an effective heat of combustion of 19.2 MJ/kg were identified as an appropriate substitute.

The typical moisture content of fecal sludge treated by operating BRs is 35% moisture on a mass basis, i.e. 35% of the final feedstock mass entering the BR consists of water and 65% of dry fecal sludge. To simulate this moisture content, wood pellets were mixed with the appropriate mass of water in a large tumbler until the water was fully absorbed. The resulting mixture had the consistency of wet sawdust and could be smoothly fed into the BR.

### Thermal power balance test

The thermal power balance testing was conducted on the BR with the ORC disconnected. A heat sink was connected to the oil loop to simulate the thermal energy removed from the system by the ORC. During operation, the BR moves through several operational modes before converging on steady-state. In real world operation, the ORC only generates power during the steady-state. To best simulate this, the BR was brought to steady-state and allowed to run for approximately three hours.

Thermal energy enters the BR through the supplied feedstock which is pyrolyzed and then combusted, releasing most of its energy as

$$Q_{in}=\Delta H_{c,fs}{\dot{m}}_{fs}\;(\mathrm{Equation 1})$$

During steady-state, the energy entering the BR through the feedstock is equal to the energy that leaves the BR. This thermal power balance may be given as

$$Q_{in}=Q_{biochar}+Q_{OHX}+Q_{HHX}+Q_{loss}\;(\mathrm{Equation 2})$$

where *Q _biochar_* is the thermal power leaving through extracted biochar, *Q _OHX_* is the thermal power extracted transferred to the working fluid by the oil working fluid heat exchanger, *Q _HHX_* is the thermal power transfered to water by the hydronic heat exchanger, and *Q _loss_* is the remaining thermal power lost to either radiant and convective heat transfer out of the exterior or jacket of the BR or through the hot gases escaping the stack.

The thermal energy remaining in the biochar is energy that was not extracted through complete combustion of the feedstock. The average thermal power lost to the biochar is determined from the biochar production rate, *ṁ _biochar_*, and the effective heat of combustion of the biochar, *ΔH _c,biochar_*, as

$$Q_{biochar}=\Delta H_{c,biochar}{\dot{m}}_{biochar}\;(\mathrm{Equation 3})$$

The total biochar produced during thermal power balance testing was collected, weighed and divided by operating time to determine a biochar production rate. The effective heat of combustion of biochar from wood pellets can be estimated as 32.3 MJ/kg ( Yang *et al*., 2017). This is similar to the average heat of combustion of biochar made from feces, 30.7 MJ/kg ( Myers *et al*., n.d.).

Thermal power extracted by the oil working fluid heat exchanger is given as

$$Q_{OHX}={\dot{V}}_{oil}\rho_{oil}c_{p,oil}(T_{oil,out}-T_{oil,in})\;(\mathrm{Equation 4})$$

where ${\dot{V}}_{oil}$ is the volumetric flow rate of oil, *_oil_* is the density of oil, *c _p,oil_* is the specific heat of the oil, and *T _oil,out_* and *T _oil,in_* are the temperatures of the oil working fluid entering and leaving the oil heat exchanger. The oil used in the BR CHP oil working fluid heat exchanger was Duratherm FG, whose thermal are seen in Table 1.

**Table 1.** Thermal properties of Duratherm FG oil working fluid.

Temperature (°C)	Density (kg/m ^3^)	Heat Capacity (kJ/kg • K)
25	853.30	1.93
45	839.80	1.99
65	826.40	2.06
85	812.90	2.12
105	799.50	2.19
125	786.02	2.25
145	772.56	2.32
165	759.11	2.38

The majority of this thermal power is transported to the ORC where it is used in electrical generation. Some thermal power is lost through so called “pipe heat losses”, where thermal power being transported in the fluid is lost through conduction to the pipe walls carrying the fluid. These pipe heat losses can be estimated as

$$Q_{PHL}=(T_{out}-T_{amb})/(R_{pipe}+R_{insul})\;(\mathrm{Equation 5})$$

where *T _amb_* is the ambient air temperature, *R _pipe_* is the effective thermal resistance for the pipe leading from the oil working fluid heat exchanger to the ORC and *R _insul_* is the effective thermal resistance of the insulation wrapping the pipe. The effective thermal resistance for a pipe or hollow cylinder is determined as

$$R=ln(r_{o}/r_{i})/(2*\pi *L*k)\;(\mathrm{Equation 6})$$

where *r _o_* is the outer radius, *r _i_* is the inner radius, *L* is the length, and *k* is the thermal conductivity of the material.

Thermal power is extracted by the hydronic heat exchanger is given as

$$Q_{HHX}={\dot{V}}_{w}\rho_{w}c_{p,w}(T_{w,out}-T_{w,in})\;(\mathrm{Equation 7})$$

where ${\dot{V}}_{w}$ is the volumetric flow rate of water, *_w_* is the density of water, *c _p,w_* is the specific heat of the water, and *T _w,out_* and *T _w,in_* are the temperatures of the water entering and leaving the hydronic heat exchanger.

The remaining thermal power leaves the system through jacket and stack losses. The total of the jacket and stack losses are calculated using Equation 2 when the other elements of the thermal power balance are known. Jacket losses consist of thermal power convected or radiated away from the exterior of the BR. Stack losses include all thermal power in the hot exhaust gases leaving the system. Jacket temperatures were measured with an infrared thermometer, once every 30 minutes, at four points on each module of the BR: the pyrolysis-combustion module, the oil working fluid heat exchanger module, the hydronic heat exchanger module. Average temperatures were used to calculate total radiative and convective heat transfer from each module of the BR. The exterior of the BR is not a uniform temperature, adding significant uncertainty to calculations of heat transfer from the surface. Further, precise pressure and flow measurements were not taken, limiting the ability to explicitly calculate energy flow in the escaping hot gases. Instead, these losses were lumped together.

During steady-state operation, water, oil and air temperatures of gases were recorded using the following thermocouples and thermistors: stack temperature (Thermocouple Omega Engineering KQIN-18U-6), catalyst temperature (Thermocouple Omega Engineering CAIN-18U-24-NHX), pyrolysis temperature (Thermocouple Omega Engineering CAIN-18U-18-NHX), water and oil temperature (Thermistor QTIQTIP68-14F-96). The flow rate oil was measured using Omega FDT-21 ultrasonic flow meter. The flow rate of water was measured using Omega FTB-30 flow meter. Power draw of the BR CHP system was measured using a WattNode Pulse energy and power meter (Continental Controls Systems, LLCWNB-3Y-400-P). The meter measures energy using a current transformer clamped around the mains power cable for the BR CHP system connected to the wall-outlet. Although thermal energy is provided by the BR for drying incoming feedstock, the dryer itself is not part of the BR CHP system as defined in the ISO/PC 318 standard.

These sensors were integrated with the BR CHP controller with the results recorded in real time using Biomass Controls kelv°n (v1.3.1) data management infrastructure, a proprietary software, which could be applied to any faecal sludge treatment system that uses sensors for digital data collection. Measurements were time-averaged across steady-state operation. Additional measurements, such as feedstock input rate, biochar production rate, and jacket temperatures were measured approximately every 30 minutes throughout the test. Data gathered is available as Underlying data ( Schoebitz, 2019)

### Data management

Collected data is stored in a standard relational cloud database that provides a flexible and dynamic data management platform ( Schoebitz *et al*., 2018). Open source data science tools were used for data analysis to increase reproducibility, collaboration and communication ( Lowndes *et al*., 2017). R Statistical Software v3.5.1 and the RStudio Integrated Development Environment v1.2.1206 were used for data analysis ( R Core Team, 2018; RStudio Team, 2018). The following open source R packages were used to perform data evaluation from initially accessing data to producing the final manuscript: bookdown v0.9, DBI v1.0.0 , dbplyr v1.2.2, here v0.1, hms v0.4.2, lubridate v1.7.4, networkD3 v0.4, purrr v0.3.0, RMySQL v0.10.15, rstudioapi v0.8, snakecase v0.9.2, stringr v1.4.0, tidyverse v1.2.1 ( Allaire *et al*., 2018; Allaire *et al*., 2017; Grosser, 2018; Grolemund & Wickham, 2011; Henry & Wickham, 2019; Müller, 2017; Müller, 2018; Ooms *et al*., 2018; R Special Interest Group on Databases (R-SIG-DB) *et al*., 2018; Wickham & Ruiz, 2018; Wickham, 2019; Wickham, 2017; Xie, 2016).

### Reproducibility

For this study we have used a Biomass Controls Biogenic Refinery and kelv°n software, proprietary equipment and software.

The results presented can be reproduced using any thermochemical fecal sludge treatment unit, however, careful consideration to how much energy is released in different treatment processes would be needed to generalize the results.

Kelv°n data plotter can be integrated with any set of sensors and is freely available to download.

## Results

### Thermal power balance

**Figure 2.**
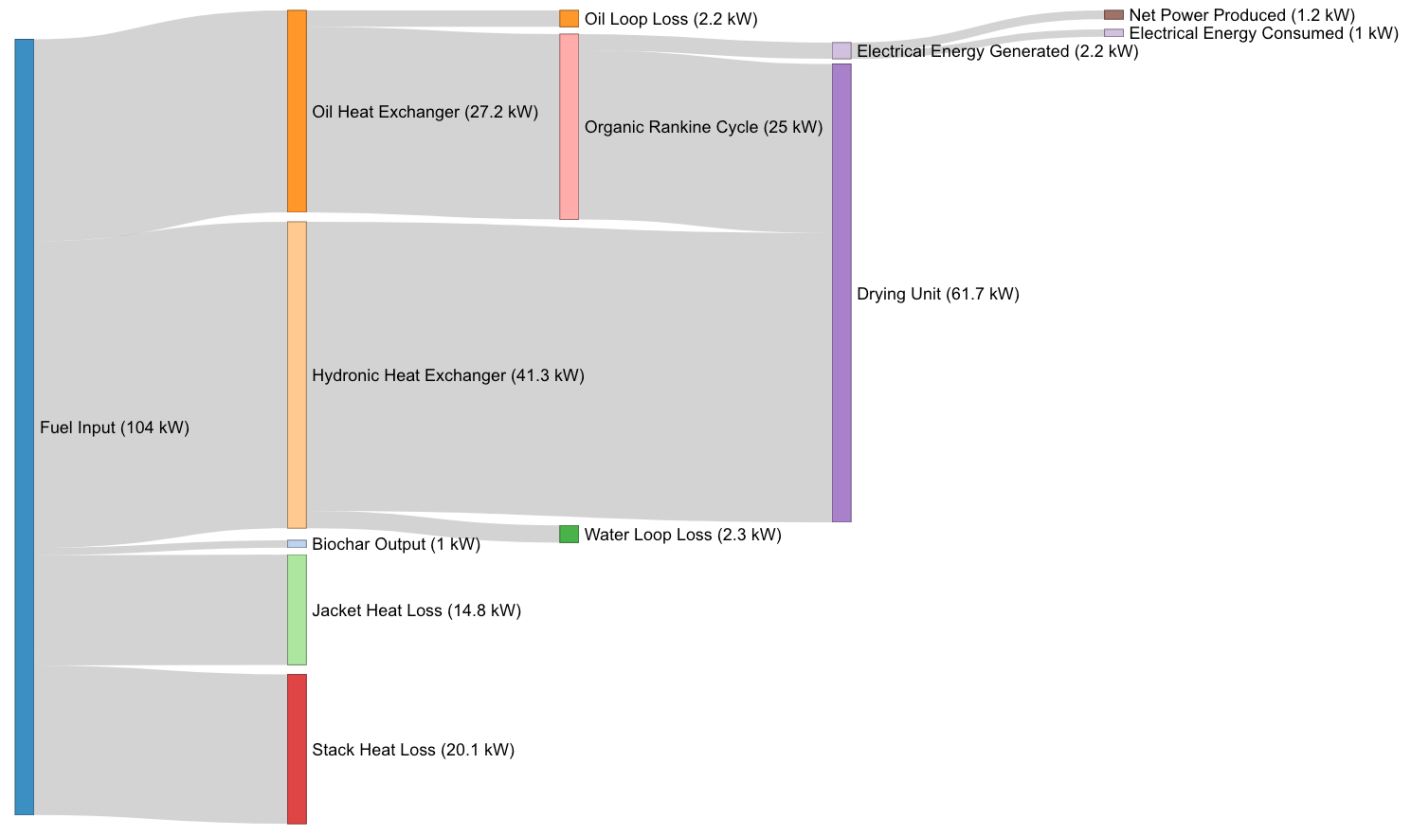
A Sankey diagram of energy flow of the Biogenic Refinery Combined Heat and Power unit during steady-state operation.

A thermal power balance in the Biogenic Refinery was constructed using the aforementioned measured system temperatures and flow rates. This thermal power balance can be seen in Figure 2. Specific details on the thermal power calculations for each element follows.

### Feedstock input

The surrogate feedstock described above was fed steadily into the BR. The fuel feed rate was measured to average 21.1 kg/hr on a dry basis. The calorific value of the feedstock was 19.2 MJ/kg on a dry basis, resulting in a total heat input of 112.5 kWth. Of this, 8 kWth were necessary to evaporate the water contained in the fuel prior to combustion. The remaining 104.5 kWth were released into the system.

### Biochar output

An average of 0.108 kg/hr of biochar was produced during operation. The biochar has an estimated caloric value of 33.8 MJ/kg. Therefore, 1 kWth of the original 104.5 kWth released into the system were not combusted and removed as biochar.

### Oil working fluid heat exchanger

The oil working fluid heat exchanger circulated Duratherm FG oil through the BR with an average flow rate of 12.7 ± 1.9 L/min, as measured by an ultrasonic flow meter. The average measured temperature of oil coming into the system was 88.2 ± 4 °C and the average measured temperature of oil entering the ORC was 153.5 ± 9.3 °C, both measured with thermistors connected to the BR controller.

Using known thermal properties of the chosen Duratherm oil, it can be calculated that 25 kWth of power were delivered from the system. The thermal energy in the oil loop was continuously transferred to a heat exchanger as part of the ORC system, where a portion of this thermal energy was subsequently converted into electricity. An additional 2.2 kWth were extracted by the oil heat exchanger, but were dissipated as “loop losses”, or thermal energy losses through the oil pipe assembly into the ambient air.

### Hydronic heat exchanger output

The hydronic heat exchanger circulated water through the BR with a measured flow rate of 13.8 ± 6.6 L/min. The average measured temperature of water coming into the system was 36.3 ± 7.7 °C and the average measured temperature of water exiting the system was 81.7 ± 5.4 °C .

Using known thermal properties of water, it can be calculated that 38.9 kWth were delivered by the hydronic heat exchanger. This thermal energy was continuously dissipated through a radiator to simulate a connected, upstream fuel drying system. An additional 2.3 kWth were extracted by the hydronic heat exchanger, but were dissipated as “loop losses”, or thermal energy losses through the water pipe assembly into the ambient air.

### Jacket and stack losses

Jacket and stack heat losses compose the remainder of the unaccounted for power leaving the system. A total of 14.8 kWth account for jacket heat losses and the remaining 20.1 kWth are estimated to be stack heat losses. The pyrolysis-combustion module had a measured average surface temperature of 141.5 °C. The result is an estimated jacket heat loss of 7.1 kWth, of which 2 kWth were due to convection and 5.2 kWth due to radiation. The oil working fluid heat exchanger module had an average surface temperature of 130.2 °C. The result is an estimated jacket heat loss of 5.7 kWth, of which 1.3 kWth were due to convection and 4.5 kWth due to radiation. The hydronic heat exchanger had an average surface temperature of 68 °C. The result is an estimated jacket heat loss of 7.1 kWth, of which 0.7 kWth were due to convection and 1.3 due to radiation kWth.

The ambient air temperature was 25.6 ± 0.5 °C. The average temperature leaving the pyrolysis-combustion module was 880.8 ± 28.1 °C. This temperature exceeds IWA 28:2018 temperature threshold requirement for pathogen free outputs. The average temperature leaving the oil working fluid heat exchanger module was 210.3 ± 64.1 °C. The average temperature leaving the hydronic heat exchanger module, or the entire BR, was 110.6 ± 4.4 °C.

Table 2 shows the complete thermal power balance. Out of 104.5 kWth that went into the BR in the form of fuel, a sum of 64.9 kWth were captured as thermal power output, while a total of 39.5 kWth can be accounted as power losses.

**Table 2.** Final power balance.

Parameter	Thermal Power (MJ/kg)
Fuel Input	104.5
Biochar Output	1.0
Oil Heat Exchanger	25.0
Oil Loop Loss	2.2
Hydronic Heat Exchanger	38.9
Water Loop Loss	2.3
Jacket Heat Loss	14.8
Stack Heat Loss	20.1

**Figure 3.**
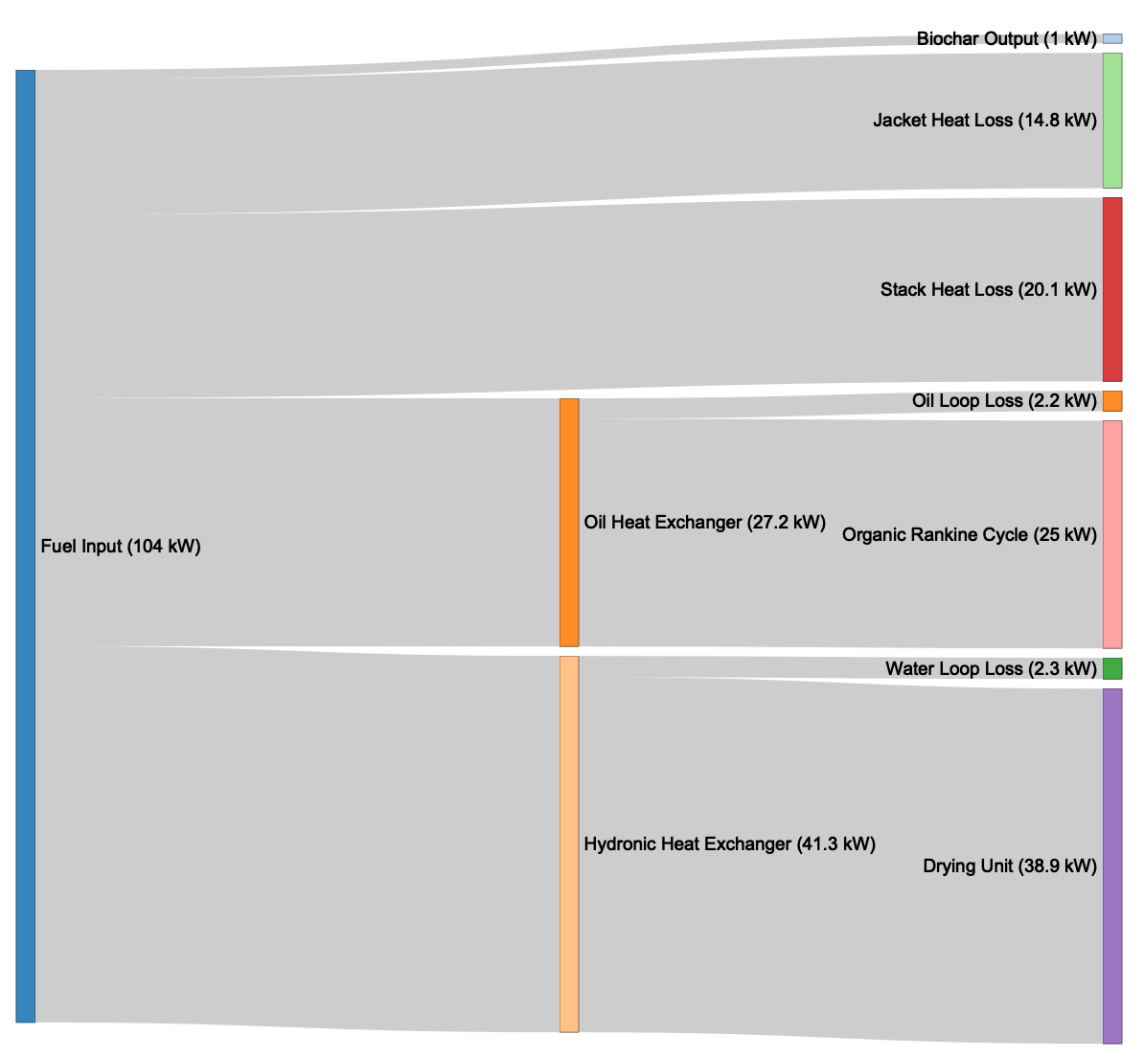
Thermal power balance of the Biogenic Refinery.

The thermal power balance is visualized as a Sankey Diagram in Figure 3.

### Electrical power balance

During steady-state operation of the Biogenic Refinery, the ORC generator was engaged and allowed to produce power. The ORC received approximately 25 kWth of power from the oil working fluid heat exchanger. This was converted into approximately 2.2 kWe of electrical power, an efficiency of 8.8 %, slightly exceeding the nominal 8% efficiency of the ORC. For the purposes of this test, the electrical power was released through a heating element and not used to power the system or charge a battery.

**Figure 4.**
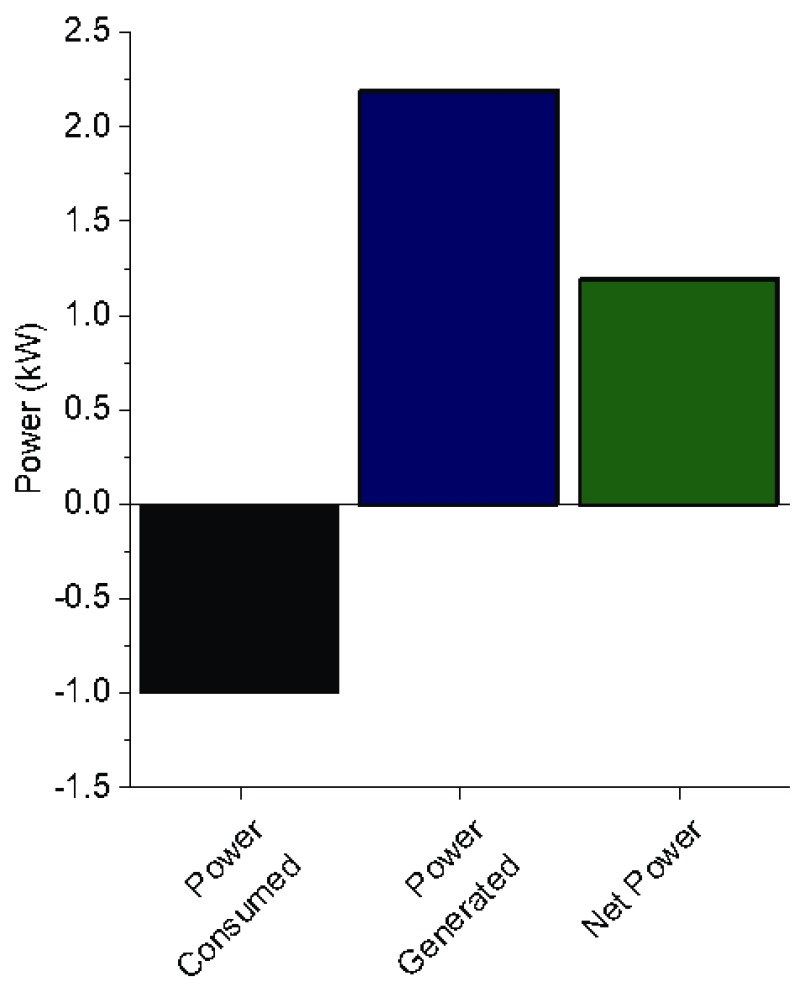
Electrical power balance of the Organic Rankine Cycle.

The average power draw from the BR CHP system during steady-state was 1 kWe. Figure 4 shows the power consumed by the CHP system compared to the power generated by the ORC during steady-state operation. The BR CHP system produced 1.2 kWe net power as the calculated difference between these terms.

## Conclusions

The present study demonstrated that the BR CHP system is capable of simultaneously meeting two key criteria of the emerging standard for community scale FSTU, ISO/PC318. Inactivation of pathogens was fast and consistent. The average treatment temperature of the BR ensures pathogen free outputs exceeding the CFR 40 Part 503 and emerging standard ISO/PC318 pathogen threshold requirements, though this will have to be demonstrated through the compliance testing required by the ISO/PC 318 standard ( US EPA, 2018). Further, the energy contained in the fecal sludge can be effectively utilized through thermal oxidation, freeing it for use to meet the electrical needs of the FSTU. The BR CHP system produced an electrical energy surplus of 1.2 kW.

This demonstration does not fulfil all of the requirements of the emerging standard. In addition to this energy positivity, a true energy independent system needs suitable energy storage and management techniques. Further, system safety, reliability, and usability, and pathogen output levels must all be verified through official certification testing, upon completion of the emerging standard. While all of these will be the subject of future efforts, the present work shows promise for a near term solution to the global need for community scale waste management.

## Data availability

### Underlying data

Zenodo: larnsce/chp-article: Second draft release. http://doi.org/10.5281/zenodo.2562836 ( Schoebitz, 2019)

This project contains the following underlying data –

- Raw (folder containing raw data for all calculations)
- chp_article_data_tables_metadata.md (Metadata for all data tables)

Data are available under the terms of the Creative Commons Zero “No rights reserved” data waiver (CC0 1.0 Public domain dedication).

## Software availability

R Markdown manuscript showing underlying source code for calculations. https://github.com/larnsce/chp-article/blob/master/manuscript/chp_research_article_manuscript.Rmd

Archived source code: http://doi.org/10.5281/zenodo.2562836 ( Schoebitz, 2019)

License: Creative Commons Zero "No rights reserved" data waiver (CC0 1.0 Public domain dedication)
