# Supplementary material for: Towards an off-grid fecal sludge treatment unit: demonstrating energy positive thermal treatment
Source: Gates Open Res. 2019 Apr 10;3:1176. [Version 1] doi: 10.12688/gatesopenres.12929.1 (PMC6733003; doi:10.12688/gatesopenres.12929.1)
Supplement: Peer review history

# Peer review: Reviewer response for version 1

**DOI:** 10.21956/gatesopenres.14031.r27705

**Published:** 2019-09-06

**Authors:** Bryan W. Weber

**Affiliations:** 1 Department of Mechanical Engineering, University of Connecticut, Storrs, CT, USA

The authors describe a new system for treatment of waste products, focusing on fecal sludge. The system is demonstrated to produce electrical power in excess of its needs during steady state operation using an organic Rankine cycle. In general, the paper is well-written, and the results are important and relevant to the community. I have several concerns about the presentation of the methods and results and some of the calculations could use more explanation, as detailed below.

**Introduction, third paragraph:**

- The authors presuppose the conclusion of their study here, which is inappropriate at this point. It would be better to say that the objective of the present study is to determine whether such an FSTU system is capable of producing neutral or excess power and leave the conclusion about this hypothesis until the conclusions section.

 

**Methods:**

- I find the description of the operation of the system to be very confusing and Figure 1 does not clarify the system sufficiently. Where on Figure 1 is the pyrolysis/combustion occurring? Where are the stack gases directed? Do they go through the oil heat exchanger first, and then through the hydronic system? The authors should label each working fluid in the loops on the diagram. In addition, I do not see the hydronic heat exchanger module on the figure. What are the “Dryers” on the figure?
- The authors should state typical pressure and temperature values in their work of the oil, water, and ORC working fluids.

**Description of ORC:**

- The authors state “the heated water is used for heating” but do not specify heating of what. What is the water heated by the rejected energy from the ORC used to heat? I believe the authors clarify at the end of the paragraph that the ORC cooling water is combined with the hot water from the hydronic system for drying, but it is not clear.
- Other questions about the ORC overall: What is the working fluid in the ORC? Who is the manufacturer? Is it an off-the-shelf component or custom-built? How is the power output controlled? Is the listed generation capacity the net or gross capacity (does it account for the pump work)?
- I find the discussion of the ORC nominal efficiency to be confusing. This may be because I am not clear about the distinction between the energy transfer from the oil to the ORC working fluid and from the ORC working fluid to the cooling water in this paragraph. My understanding from reading the paragraph is that the hot oil is used to heat the ORC working fluid, and all of the energy from the oil is transferred to the ORC working fluid. The ORC uses 8% of this energy to produce electrical power output, and the remainder can be used for other purposes.
- If this is correct, I’m curious why the efficiency of the ORC is so low. If the temperature of the working fluid is high enough to support further heat transfer after expansion, it seems that additional electrical energy could be extracted. If the temperature of the working fluid after the expansion is relatively low, then it cannot be used for heat transfer. Clarifying this paragraph would significantly help understanding the results, so I think the authors should revise this paragraph substantially.

 

**Feedstock:**

- The authors should state how the heating value of the wood pellets is determined and give an uncertainty on the measurement if possible. What is the “appropriate mass of water”? How is the water content of the wood pellets prior to mixing determined? How is the water content of the wet sawdust mixture confirmed?

**Thermal power balance test:**

- What sort of heat sink was used to replace the ORC? How much power did it absorb compared to how much it is rated to absorb? Was the energy balance of this component checked?
- How is the system brought to steady state? What criteria are used to determine that the system is operating at steady state? The authors could show a typical time-history plot of key measured variables in the system to demonstrate the phases of operation and when the system reaches steady state.
- Eq 1: Typically, symbols that represent power values have a dot over the symbol. I find the phrase “Q_OHX is the thermal power extracted transferred to the working fluid by the oil working fluid heat exchanger” to be very confusing. Which working fluid of which component? Why is working fluid repeated twice? The authors should clarify what is meant here, and try to replace the phrase “oil working fluid heat exchanger” where it appears elsewhere.
- Eq 4: Given that both density and specific heat are functions of temperature, this equation should be an integral with respect to temperature. How did the authors handle these variations? The phrase “whose thermal” should be “whose thermal properties”.
- The sentence prior to Eq. 7 has an extra “is”. Same comment about density and specific heat being functions of temperature as for Eq. 4.
- The sentence “During steady-state operation, water, oil and air temperatures of gases” does not make sense to me. Can the authors clarify? The authors should label the location of each thermocouple/thermistor on Fig. 1.
- “Although thermal energy is provided by the BR for drying incoming feedstock”: Is the feedstock dried to lower than 35% moisture content? If so, how low?

 

**Results:**

- Overall in the results section, the authors are inconsistent about indicating uncertainties in measured and calculated quantities. They should present uncertainties for all of these quantities in the article, and also describe how the uncertainties are estimated or calculated.

 

**Thermal power balance:**

- The Sankey diagram in Fig. 2 includes electrical energy output, but the authors stated that the ORC was taken out of the loop for the thermal power balance test. This seems inconsistent and the authors should please clarify. The authors note in the text that the fuel input power is 104.5 kW but only show 104 kW on the diagram, although other numbers are specified to one decimal place. The authors should be consistent.

 

**Feedstock input**:

- The authors should explain how the number of 8 kW was calculated for the evaporation of water.

 

**Biochar output:**

- The authors previously stated that the heat of combustion of biochar is 32.3 MJ/kg, but here they list 33.8 MJ/kg as the caloric value. Which is correct, and which was used?

 

**Oil working fluid heat exchanger:**

- How did the authors estimate that 2.2 kW were lost in the oil loop? Are these the pipe losses discussed previously? In that context, they were mentioned only for the pipes carrying ORC working fluid.

 

**Jacket and stack losses:**

- The authors previously stated that jacket and stack losses would be lumped together due to difficulty in measuring or estimating the required temperatures and flow rates. However, they are separately calculated in this section. How was this done?

 

**Table 2 and Fig. 3:**

- What information is present in this table and figure that isn’t present in Fig. 2?

 

**Electrical power balance:**

- How is the power draw of the system calculated? Does it include all the fans, pumps, electronics, and other accessories necessary to run the system, including the oil, water, and working fluid pumps? I don’t think that Fig. 4 adds anything to the manuscript and could be removed.

I confirm that I have read this submission and believe that I have an appropriate level of expertise to confirm that it is of an acceptable scientific standard, however I have significant reservations, as outlined above.

# Peer review: Reviewer response for version 1

**DOI:** 10.21956/gatesopenres.14031.r27702

**Published:** 2019-09-06

**Authors:** Thomas A. Trabold

**Affiliations:** 1 Golisano Institute for Sustainability, Rochester Institute of Technology, Rochester, NY, USA

This paper presents an analysis of a hybrid pyrolysis-combustion system applied to treatment of fecal sludge. I believe the work provides a meaningful contribution to the literature and is suitable for indexing. The comments below are offered to improve the quality of the final manuscript.

- More details need to be provided on the operation of the Biogenic Refinery and the integration of the organic Rankin cycle (ORC).
   
  - Include a more detailed description of the mechanization of the Biogenic Refinery. For example, why is the system operated as a combined pyrolysis (oxygen free) stage followed by a combustion stage, instead of a single-stage gasification system that would run at higher temperature and provide roughly the same biochar yield?
  - Explain why the ORC is the best energy conversion device. Why is utilizing thermal energy from combustion of syngas generated in the pyrolysis stage in an 8% efficient ORC better than generating syngas that can be converted to electricity at an efficiency of between ~25% (ICE genset) and ~60% (solid oxide fuel cell). If cost was the primary consideration, this should be stated so.
- In the section on “Description of the ORC electrical generation” include more details on the ORC device, such as the manufacturer and system specifications. I agree with one of the earlier reviewers (Septien Stringel, 2019 ^1^) that combining a biomass thermochemical conversion system with renewable energy generation to make the integrated system essential net energy positive is quite unique and needs to be emphasized.
- Editorial:
   
  - Abstract: change “criteria” to “criterion”.
  - Keywords: add “biochar” and “pyrolysis”.
  - Both “fecal” and “faecal” are used, but need to be consistent throughout the manuscript.
  - Page 3, right column: change “inactive” to “inactivate”.
  - Page 3, right column: include “NY” after “Lewiston”.
  - Page 5: text under Equations 4 and 7 should change the order of “leaving” and “entering”.
  - Page 5, left column: replace “…Duratherm FG whose thermal…” with “…Duratherm FG whose density and heat capacity…”.
  - Page 5, right column, bottom paragraph: insert “of” in the phrase “flow rate oil”.

I confirm that I have read this submission and believe that I have an appropriate level of expertise to confirm that it is of an acceptable scientific standard.

## References

[1] : Peer Review Report For: Towards an off-grid fecal sludge treatment unit: demonstrating energy positive thermal treatment [version 1]. *Gates Open Research*.2019;3(1176) : Reference source 10.12688/gatesopenres.12929.1PMC673300331508581

# Peer review: Reviewer response for version 1

**DOI:** 10.21956/gatesopenres.14031.r27057

**Published:** 2019-05-08

**Authors:** Swaib Semiyaga

**Affiliations:** 1 Department of Civil and Environmental Engineering, College of Engineering, Design, Art and Technology, Makerere University, Kampala, Uganda

I would like to thank the authors for this well written paper. This topic is very relevant, as we are faced with challenges of decentralized faecal sludge management in several urban settings. This study has demonstrated that a Biogenic Refinery (BR) together with a combined heat and power (CHP) can be operated using the energy from a feedstock (faecal sludge) to generate heat and a net positive power in addition to inactivation of pathogens in faecal sludge. The paper can be indexed, after clarification on the following few observations:

- It is a good innovation to have off-grid solutions due to the nature of several places with limited power access, yet in need of such services. However, the study has not shown whether the produced power is enough to run the FSTU.
- The moisture content of the feedstock required in BR is 35%, yet FS can be over 95% water. I understand you mentioned that the dryer unit is not part of this BR-CHP system. However, feedstock preparation by reducing moisture content from 95% to 35% requires vast amounts of energy input. I propose you further analyze the drying requirements of feedstock using the generated heat energy for drying. This is important to check the adequacy of the produced heat in meeting the feedstock drying requirements. 
- What is the reference used on 8 kWh requirement to evaporate the initial water in the feedstock? It would also be important to indicate a note that this may vary for different feedstocks.
- Also include the thermal properties of water, as you did for oil.
- There is unnecessary repetition in presentation of results. Information on Figure 3 can be got from Figure 2.

I confirm that I have read this submission and believe that I have an appropriate level of expertise to confirm that it is of an acceptable scientific standard.

# Peer review: Reviewer response for version 1

**DOI:** 10.21956/gatesopenres.14031.r27054

**Published:** 2019-04-26

**Authors:** Santiago Septien Stringel

**Affiliations:** 1 Pollution Research Group, University of KwaZulu-Natal, Durban, South Africa

This paper presents the evaluation of a thermal process technology that allows generating biochar, heat and electricity from the treatment of faecal sludge. The technology was evaluated through an energy balance where the key parameters were determined from measurements during the operation of the machine. The authors concluded that this process enables reaching high enough temperatures to ensure pasteurization of the output streams and it was demonstrated to be energy positive. Both criteria are important to achieve certification of the technology under the ISO/PC318 standards that are currently under development.

 My major critique of this work is that its originality is not emphasized enough and there is a lack of analysis of the results. As currently written, the paper looks more like a report of results than a scientific paper. The technology presented in the paper is novel because it combines a small-scale faecal sludge thermal treatment process with a unit for electricity generation. This feature is rare for the processing of faecal sludge at such a small scale. The authors should insist in this novelty that makes their technology unique, and the different sections of the papers should be re-written accordingly. On the other hand, the results section could also display the experimental results obtained during the operation of the process, and not only focus on the results of the calculations. Moreover, the results from the calculations should be analysed in a deeper level. For example, the authors could include the determination of the thermal efficiency of their process, explain the reasons of efficiency losses and propose eventual solutions to improve this. Concerning the written quality of the paper, most of the parts are clear and well-structured, nevertheless a few parts are too repetitive or confusing, and some terms are incorrectly employed.

 This paper presents a good example of an efficient thermal process technology for faecal sludge treatment that is worthy to be disseminated. Therefore, the indexing of the present paper will be useful for the sanitation community. However, the paper in its actual form needs to be improved significantly before indexing.

 I have included, here below, a list of specific comments for the improvement of the manuscript quality.

  

- General observations:
   
  - I have never heard about the term “faecal sludge treatment unit (FSTU)”. We commonly employ: “faecal sludge treatment plant (FSTP)”.
  - Instead of using the term “thermal power balance”, it will be more correct to use “heat balance” or “energy balance”.
  - The brand and model of instruments should be written in italics.
  - There are too many terms in your keywords (it should be limited to the most 5-6 relevant terms).

 

- Introduction:
   
  - General observations: There are not enough references in the introduction. You should include more references about thermal processes of faecal sludge that are currently available or are under development at the different scales, as well as the beneficiation of biochar issued from faecal sludge pyrolysis. You could also include a summary of the relevant results of investigations about faecal sludge thermal decomposition. In all this, you could include references from sewage sludge, which is a similar material.
  - Likewise, you could discuss about the different means to produce electricity in small-scale plants (as your case), and give more information about the organic Rankine Cycle and its applications. 
  - In the problem statement and presentation of the scope of your work, you have to emphasize the novelty of your technology with respect to what has been previously done.
  - Paragraph 1, line 16: You must define “energy neutral” and “energy positive”.
  - Paragraph 1, line 16-18: The explanation “operate…..sources” is incorrect. You mean that the only source of energy in the process will come from faecal sludge, without the input of any additional source of energy.
  - Paragraph 4, last sentence: You are concluding in the introduction, which is incorrect. This should be placed in the conclusion.

 

- Methods:
   
  - Title: Typically it is “Materials and Methods”.
  - Figure 1: The captions of figures are conventionally written without any conjugated verbs.
  - Section “Feedstock”, paragraph 2, first sentence: The moisture content of 35% is too low to be from a raw faecal sludge. So, I think that you are referring to previously dewatered or dried sludge. You have to specify this.
  - Section “Thermal power balance test”, equation 1: You have to introduce the variables in the text (as you have done for the other equations).
  - Section “Thermal power balance test”, paragraph 3, line 2: We cannot use the term “complete combustion” as this will imply that the biochar was completely burnt. I suggest to change “not extracted….. feedstock” to “remaining in the solid after thermal decomposition of the feedstock”.
  - Section “Thermal power balance test”, paragraph 3, line 3: The thermal energy in the biochar cannot be considered as a thermal loss, as this energy can be released in a later stage.
  - Section “Thermal power balance test”, paragraph 4, last two sentences: The values of the calorific value of the biochar seem quite high. Are they in ash-free basis?
  - Section “Thermal power balance test”, paragraph 5, last sentence: The oil has already been described. Avoid this type of repetition.
  - Section “Thermal power balance test”, paragraph 8: The explanation in this paragraph is quite confusing. It is not clear how you calculated the heat losses: did you calculate them through difference using the general heat balance and knowing the other terms, or by the corresponding heat transfer equations?
  - Section “Thermal power balance test”, paragraph 10: You must indicate how the feedstock mass flowrate was measured.
  - Section “Thermal power balance test”, paragraph 10, line 4 – 5: Avoid advertising the products from Biomass Controls (here in “which could... data collection”).
  - Section “Thermal power balance test”, paragraph 10, last sentence: There is a full stop (“.”) missing at the end of the sentence.
  - Section “Data management”, paragraph 1, line 4-5: I don’t understand how the use of open source data tools can enhance collaboration and communication.
  - Section “Reproducibility”: You don't explicitly mention how you tested reproducibility.

 

- Results:
   
  - General observations: This section should show and comment on the measurement of the different parameters of the process during its operation (example: evolution of the temperatures at the different locations as a function of time).
  - Title: Typically, it is “Results and discussion”.
  - Section “Biochar output”: You could mention the char yield, an important pyrolysis parameter.
  - Section “Oil working fluid heat exchanger”, paragraph 1, line 3: You have to mention how you measured the flowrate in the Materials and Methods, not here.
  - Section “Oil working fluid heat exchanger”, paragraph 2, line 6: Incorrect use of the word "extract". The 2.2 kWth represents thermal losses, and it is not possible to extract useful energy from it.
  - Section “Jacket and stack losses”: You have to comment on the thermal losses. Are these values high or low? Can they be reduced? How?
  - Section “Jacket and stack losses”, paragraph 1: You have to indicate in the Materials and Methods how the convective and radiative contributions in the heat losses were respectively calculated.
  - Section “Jacket and stack losses”, paragraph 2: You have to be clearer about what the “average temperatures” correspond to.

 

- Conclusions:
   
  - General observation: In the conclusion, you have to emphasize that you have proven the feasibility of your process, which presents the novelty to combine heat treatment of faecal sludge at relative small-scale with electricity production. You can also comment about the thermal efficiency of your process and how this could be improved. 
  - Paragraph 1, line 4: You cannot say that “the inactivation of pathogens was fast and consistent” without any tests.
  - Paragraph 2: The term of “energy positivity” does not exist.

I confirm that I have read this submission and believe that I have an appropriate level of expertise to confirm that it is of an acceptable scientific standard, however I have significant reservations, as outlined above.
